# Etiology, Classification, and Restorative Management of Amelogenesis Imperfecta Among Children and Young Adults: A Scoping Review

**DOI:** 10.7759/cureus.49968

**Published:** 2023-12-05

**Authors:** Saad S Bin Saleh

**Affiliations:** 1 Department of Pediatric Dentistry and Orthodontics, College of Dentistry, King Saud University, Riyadh, SAU

**Keywords:** amelogenesis imperfecta, phenotype, genotype, children, rare condition, restorative dentistry, classification, etiology

## Abstract

Amelogenesis imperfecta (AI) is a rare genetic disorder affecting children and adults. Knowledge about AI is limited to clinical representation and radiographical findings. Various treatments are provided to children with AI, yet no definitive treatment guideline has been suggested in the literature. This scoping review highlights the knowledge of the etiology and classification of AI and synthesizes these findings in a comprehensive review, focusing mainly on the various forms of AI in children and management with a restorative conservative approach. Five electronic databases, namely, PubMed, Google Scholar, Embase, Web of Science, and Scopus, were searched for the relevant articles. The search was performed in two phases: first for title and abstract, and second for full-text articles. The studies included in this scoping review were published from 2013 to August 2023. The data extraction was done on a customized sheet. A total of 33 studies were included in this review, of which 19 were reports and series, seven were observational, and seven were reviews. Most patients included in this review suffered from the hypoplastic type of AI (54%), followed by hypomatured (36%), and hypocalcified (10%). The treatment modalities explained were divided into the following three phases: temporary, transient, and permanent. Almost all included reports suggested the requirement for guidelines for treating AI among young children. This scoping review suggests the need for guidelines for treating AI in children. Moreover, pediatric dentists should prioritize early diagnosis and treatment and long-term follow-up for AI in children to effectively enhance the patient’s psychological well-being and overall quality of life.

## Introduction and background

Amelogenesis imperfecta (AI) is a clinically and genetically heterogeneous disorder that affects the development of enamel [1]. This condition leads to the loss of enamel structure, composition, and amount, resulting in thinning, sensitivity, and aesthetically unpleasant teeth. AI is present in both primary and permanent dentition; even if not seen macroscopically, it varies in phenotypes [2]. The prevalence of AI varies from 1:700 to 1:14,000 cases, depending on the population of interest [3]. Affected teeth exhibit yellow to brown discoloration, increased susceptibility to dental caries, calculus deposition, attrition, gingival hyperplasia, and often an anterior open bite. AI is caused by gene mutations (*ENAM*, *AMEL*, *DLX3*, and *P63*), which are crucial for enamel formation [4].

AI is classified into three types based on the enamel pattern and the stage at which teeth are affected. First, hypoplastic AI involves teeth with an inadequately formed enamel matrix, although the enamel is normally mineralized [5]. Second, hypocalcified AI involves teeth that are normally formed but with low-density soft enamel, making them more prone to fracture. Third, hypomaturation AI initially has the proper amount of enamel, which is calcified to a certain extent, but the enamel crystals are affected, resulting in soft enamel with a tendency to fracture [6]. AI is not associated with any systemic disease. Over the years, various classifications have been proposed, and in recent years, the classification has become more descriptive and involves molecular defects [7].

AI’s genetic etiology is caused by gene mutations, which affect the matrix protein-20, amelogenin, enamelin, and kallikrein-4 [8]. These gene sequences affect the formation of the enamel matrix, leading to thin and hypoplastic enamel, or if the defect is during the maturation stage, hypomature enamel is formed [9]. Along with enamel defects, AI is also associated with malocclusion, delayed eruption, attrition, anterior open bite, and deviant morphology [10]. Patients with AI require multidisciplinary treatment from an early age. Various treatments (restorative, prosthetic, and orthodontic) have shown a positive outcome. However, close follow-up is required to maintain the treatment outcome.

The treatment of AI follows four stages, namely, emergency, prevention, stabilization, and definitive treatment [6]. Treating pediatric patients with AI is challenging for the dentist as it involves handling a young child and performing extensive and demanding multiple-visit treatment [8]. Moreover, preteen children also need psychological support to develop a coping strategy [7,8,11]. Additionally, the investigation and treatment of AI depend on various factors, such as clinical complaints, psychological issues, and restorative challenges. AI patients have various clinical issues, such as poor oral hygiene, chronic gingivitis, caries, discoloration, and short crowns. Most pediatric patients report complaints of multiple caries and sensitivity to their dentists [12].

The treatment of primary dentition consists of restorative management and the placement of a stainless-steel crown on posterior teeth to prevent decay. For mixed dentition, long-term temporary treatment is required for hypersensitivity, tooth substance loss, and aesthetics. The recommended long-term treatment plan for patients with AI is the placement of porcelain crowns. Although clinical use of temporary restoration is common today, recently published studies have highlighted the importance of permanent crown therapy in early adolescence without severe complications [11,13,14].

Anecdotally, AI is linked to discoloration of teeth, anterior open bite, caries, and chronic gingivitis, but this association and its impact on children and treatment planning has not been well-documented. Moreover, due to the lack of prevalence of AI cases, the knowledge regarding treatment planning of pediatric patients is limited. There is a lack of clinical burden of this disease among pediatric dentists and other specialists [13,15,16]. Additionally, there are no proper treatment guidelines for this condition among children. This leads to challenges in patient service planning and treatment delivery. Therefore, this scoping review aims to highlight the knowledge of the etiology and classification and synthesize these findings in a comprehensive review, focusing mainly on various forms of AI in children and management with restorative treatment.

## Review

Methodology

This scoping review followed the Preferred Reporting Items for Systematic Reviews and Meta-Analyses Extension for Scoping Reviews guideline and modified scoping review guidelines by Arksey et al. [17] and Munn et al. [18].

Research Question

The primary research question was “What is known from the existing literature about the etiology, classification, and restorative treatment guidelines of AI among children and young adults?” The PCC designed for this research question was P = children suffering from various types of AI; C = clinical and radiological diagnosis and genotypes; and C = clinical restorative management of AI in children. The research sub-questions were “What are the different types of AI?”; “What are the etiological characteristics of AI?”; “What are the clinical and radiological findings of AI?”; “What are AI guidelines and restorative treatment options for children and young adults?”

Search Strategy

Five databases, namely, PubMed/Medline, Scopus, Google Scholar, Embase, and Web of Science, were searched to retrieve the included articles. Additionally, reference chasing was performed to check the availability of data. Observational studies, case reports, case series, and randomized control trials published in peer-reviewed journals from 2013 until August 2023 were included. The search initially focused on the title and abstract to extract data related to AI. The search strategy was appropriately formed according to the databases. The search terms, MeSH terms, and Boolean operator alone or in combination used to extract the articles included “amelogenesis imperfecta,” “AI,” “children,” “pediatric dentistry,” “restorative treatment,” and “management.” References were managed by Endnote 20 (Clarivate, NY, USA).

Inclusion and Exclusion Criteria

This scoping review included articles related to the objectives of this study. The included participants were children aged three to 14 with a confirmed diagnosis of AI. Letters to editors, gray literature, articles, reviews published before 2013, and articles on adult AI patients were excluded.

Study Selection

Articles selected from each database were charted on Microsoft Excel (Microsoft Corp., Redmond, WA, USA). The investigator performed the study selection and screening of the title and abstracts carefully with the help of a librarian. The following peer-reviewed article types published in the English language were screened: observational studies (case-control, cross-sectional), clinical trials, systematic reviews and narrative reviews, case reports, and case series. A manual search of articles in references was also performed. Articles were retrieved for full-text reading if they met the inclusion criteria or when there was uncertainty. The investigator did this at one-month intervals to check the intraclass reliability, and the kappa statistic was calculated at a 95% confidence interval.

Data Extraction

The investigator performed the data extraction process using a predesigned standardized form. The following customized sheets were utilized for data extraction for case reports and series: (1) author, country, and year; (2) study design; (3) number of cases; (4) type of AI; (5) treatment modalities (mainly restorative); and (6) results. The customized data sheet created for observational studies included (1) author, country, and year; (2) study design; (3) number of cases; and (4) main findings. The customized sheet for review articles included information about (1) author, country, and year; (2) review/systematic review; and (3) main findings.

Synthesis of Findings

A narrative synthesis of the findings from the included studies was performed. Results were structured according to the themes in the following manner to provide a coherent summary of the findings: (1) characteristics of included studies; (2) etiology of AI; (3) classification and types of AI; and (4) restorative and esthetic management for children, along with guidelines wherever applicable.

Results

**Figure 1:**
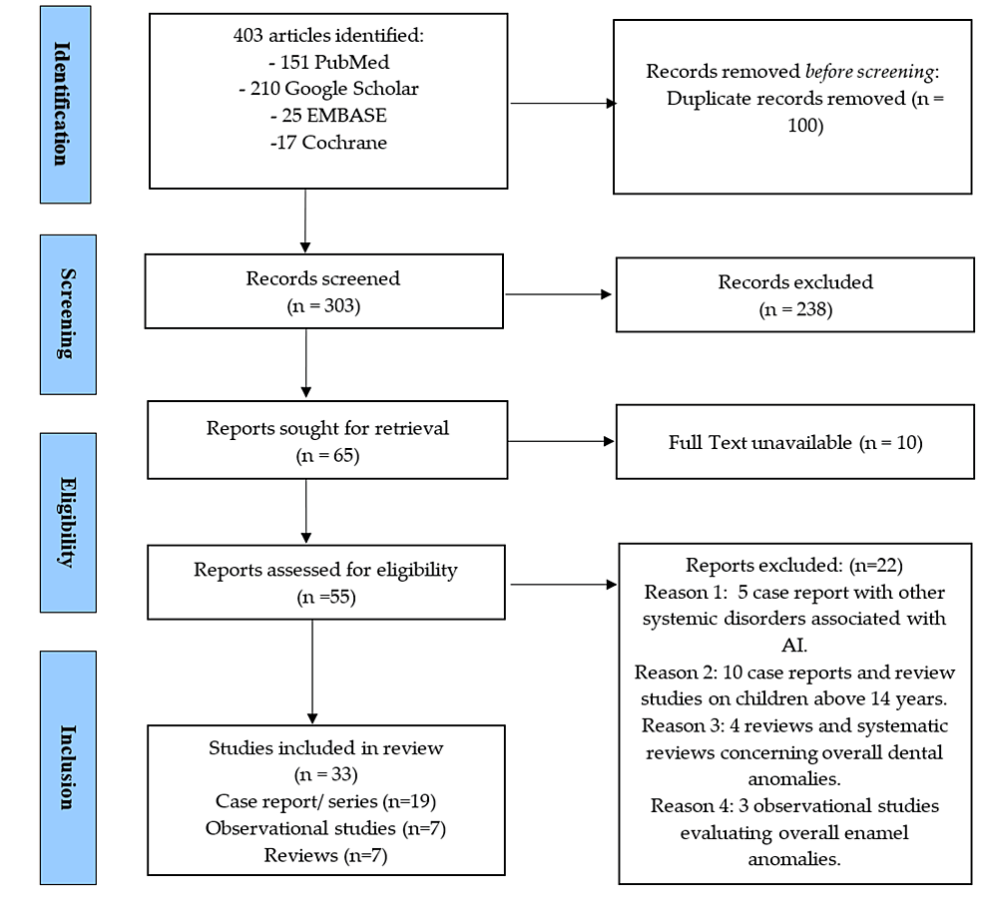
PRISMA-ScR flowchart. PRISMA-ScR: Preferred Reporting Items for Systematic Reviews and Meta-Analyses Extension for Scoping Reviews

A total of 403 articles were extracted from four different electronic databases and exported to Endnote 20 (Clarivate, NY, USA). After removing duplicates, 303 articles were retrieved for title and abstract screening, of which 238 were removed. A total of 65 articles were sought to be retrieved, of which 10 were in different languages. Finally, 55 articles were extracted for full-text reading, of which 33 articles (19 case reports/series, seven observational studies, and seven reviews) were included in this scoping review (Figure 1). The intraclass reliability was excellent for article selection (0.88) and data synthesis (0.91) after one month. Table 1 demonstrates the genotypes, phenotypes, and radiographic and clinical findings of different types of AI [4]. The articles were extracted into three categories according to the study design (Tables 2-4).

**Table 1:** Classification, etiology, radiographic, and clinical findings of amelogenesis imperfecta.

AI types	Sub-classifications	Genotypes	Phenotypes	Mode of inheritance	Radiographic findings	Clinical findings
Hypoplastic (I)	IA: hypoplastic; IB: hypoplastic; IC: hypoplastic; ID: hypoplastic; IE: hypoplastic; IF: hypoplastic; IG: enamel agenesis	AMELX, ENAM, KLK4, and MMP20	IA: Pitted; IB: localized hypoplastic; IC: generalized thin; ID: diffused hypomineralization; IF: rough enamel surface; IG: hypomineralization	IA: autosomal dominant; IB: autosomal dominant; IC: autosomal recessive; ID: autosomal X-linked dominant; IF autosomal dominant; IG: autosomal recessive	Normal enamel radiopacity thickness is markedly reduced	Enamel agenesis or defect in enamel formation causes a rough surface and discoloration, and most patients have an anterior open bite
Hypomaturation (II)	IIA: hypomaturation; IIB hypomaturation; IID: snow-capped teeth	AMBN, DLX3, and TUFT1	IIA: pigmented; IIB: Hypomaturation; IID: generalized pitted	IIA: autosomal recessive; IIB: X-Linked recessive; IID: autosomal dominant	Enamel shows similar radiopacity to dentin. Normal thickness	Alteration in enamel thickness, but the consistency of enamel is normal and softer. The altered enamel has discoloration (white opaque) and can easily get fractured
Hypocalcified (III)	IIIA and IIIB: hypocalcified	FAM83H	IIIA: generalized hypoplastic; IIIB: generalized thin	IIIA: autosomal dominant; IIIB: autosomal recessive	Lower opacity of enamel compared to dentin	Enamel has normal thickness but discolored orange-yellow enamel with poorly calcified matrix
Hypomaturation and hypocalcified with taurodontism (IV)	IVA: hypomaturation-hypoplastic with taurodontism; IVB: hypoplastic-hypomaturation with taurodontism	DLX3 and SLC24A4	IVA and IVB: generalized hypoplastic; hypocalcified and pitted with taurodontism	IVA and IVB: autosomal dominant	Lower opacity of enamel compared to dentin	A mixed appearance of hypocalcification and hypomaturation enamel and taurodontism

**Table 2:** Relevant case reports/series involving children with amelogenesis imperfecta.

Author/Country/ Year	Country	Study design	Cases	Age (M/F)	Type of amelogenesis imperfecta	Restorative treatment	Result
Elfadil et al. (2023) [19]	United Arab Emirates	Case series	3	Patient 1: 12 years/ female Patient 2: 13 years/ female Patient 3: 13 years/ female	Patients 1, 2, and 3 had type I AI	Patient 1 underwent esthetic reconstruction utilizing composite restoration by the layering method. Patient 2 underwent smile designing with Inspiro composite resin build-ups. Patient 3 underwent smile designing	After three months of follow-up, all three patients were satisfied with the outcome
Herrera-Rojas et al. (2023) [20]	Lima, Peru	Case report	1	6.5 years/ male	Type I AI	Direct restorations of the upper anterior incisors, with 3M™ Filtek™ Z350 XT Color A Universal Restorative Composite	Restorative treatment at an early age depends on clinical characteristics such as pain, appearance, and the severity of the damage to the enamel structure
Elfseyie et al. (2022) [21]	Libya	Case report	1	13 years/ female	Hypoplastic AI	Direct composite restoration of anterior teeth and metal crown restoration for deciduous molars	Composite resin maintains the teeth structure and restores the esthetic appearance and the function of teeth. The patient was satisfied with the treatment outcome
Jabin et al. (2022) [22]	India	Case report	1	12 years/ female	Hypoplastic AI	Direct composite restoration of anterior teeth and metal crown restoration for deciduous molars	Composite resin maintains the tooth structure and reduces sensitivity
Möhn et al. (2021) [23]	Germany	Case report	2	Patient 1: 7 years/ female Patient 2: 12 years/ male	Patient 1: type AI Patient 2: type 2 AI	Patient 1: Adhesive build-up and stainless-steel crowns for posterior teeth. Patient 2: Restoration with dual resin cement and fissure healing along with stainless-steel crown in second permanent molar	After six months of follow-up, patients were satisfied with their aesthetics, and no TMJ-related abnormality was observed
Novelli et al. (2021) [24]	Italy	Case report	1	9 years/ female	Type I AI	Prefabricated composite veneers were used for the patient in this report (Edelweiss Veneers, Edelweiss Dentistry, Wolfurt, Austria)	Prefabricated veneers can be a valid approach because of the minimally invasive procedures involved and the higher esthetic result obtained
Amira and Imene (2020) [25]	Tunisia	case report	1	8 years/ female	Type 2 AI	Direct dental composite restorations, and full metal crown for posterior teeth	The patient was satisfied with the esthetic appearance
Asali and Almaliki (2019) [26]	Saudi Arabia	Case series	27	3–14 years	Hypomature, hypocalcified, and hypoplastic AI	Oral rehabilitation of the primary molars with stainless-steel crowns and resin-filled celluloid crowns	There is a need for long-lasting restorative solutions for AI patients
Elelmi et al. (2019) [27]	Tunisia	Case series	4	9–14 years	Patient 1: hypoplastic AI Patient 2: X-linked hypomineralized AI Patient 3: X- linked hypoplastic AI Patient 4: type 1 AI	Stainless-steel crown onlays for permanent molars, composite resin, or glass ionomer cement for primary and permanent teeth	All four patients were satisfied with the treatment
Singh et al. (2018) [28]	India	Case series	3	Patient 1: 6 years/ female Patient 2: 11 years/ male Patient 3: 12 years/ female	Patient 1: hypoplastic AI Patient 2: X-linked hypomineralized AI Patient 3: X- linked hypoplastic AI Patient 4: type 1 AI	Scaling and polishing	Patients were recommended to maintain oral hygiene and were recalled for restorative treatment after the eruption of all permanent teeth
Toupenay et al. (2018) [29]	France	Case report	3	Patient 1: 3 years/ female Patient 2: 8 years/ female Patient 3: 14 years/ female	Patient 1: hypoplastic AIH Patient 2: hypomineralized AI Patient 3: hypomineralized AI	Patient 1: direct dental composite restorations were placed (Herculite, Kerr); stainless-steel crowns for posterior teeth Patient 2: indirect resin-based composite (Premise Indirect System, Kerr) Patient 3: indirect composite (Premise Indirect System, Kerr)	The indirect and direct resin-based composites provide the best treatment options for patients with different types of AI. The advantages of this treatment are minimal invasion and aesthetic appeal to patients
Cagetti et al. (2017) [30]	Italy	Case report	2	Patient 1: 13 years/ female Patient 2: 14 years/ male	Patient 1: hypomatured type I Patient 2: hypomatured type	Patient 1: 15% HCL gel Patient 2: bleaching with Philips Zoom WhiteSpeed Light-Activated Whitening System	Indirect and direct resin infiltration with minimal invasion has been proven as the best option for treating AI in adolescents
Leevailoj et al. (2017) [31]	Bangkok	Case report	1	10 years/ male	Hypoplastic type	For anterior teeth, resin composite veneers with minimal invasion and stainless-steel crowns were given for deciduous molars and first permanent molars	After two years of follow-up, the patient was satisfied with his dental treatment outcomes
Halal et al. (2017) [32]	Lebanon	Case report	1	12 years/ male	Hypomature AI	CAD-CAM milled polymer-infiltrate ceramic for establishing aesthetics	After two years of follow-up, ceramic infiltration showed major esthetic improvement and allowed normal eruption of permanent dentition
Chafaie (2016) [33]	France	Case report	1	14 years/ female	Hypomature AI	For incisors and canines, composite resin veneers using the semi-indirect technique were done	The patient was satisfied with the aesthetic appearance
Marquezin et al. (2015) [34]	Brazil	Case report	1	5 years/ male	Hypocalcified AI	Anterior teeth restoration was done by resin-filled celluloid and stainless-steel crowns on the primary molars	The patient was recalled after four months to check the permanent dentition, and further treatment was planned accordingly
De Souza et al. (2014) [35]	Brazil	Case report	1	8 years/ female	Hypoplastic AI	Composite restoration was provided to the upper and lower permanent incisors	The direct bonded composite restoration provided excellent conservative treatment for the protection of teeth affected by AI
Yildrim et al. (2014) [36]	Turkey	Case report	3	Patient 1: 12 years/ male Patient 2: 7 years/ female Patient 3: 6 years/ male	Patient 1: hypomaturation AI Patient 2: hypoplastic AI Patient 3: hypocalcified AI	Patient 1: fluoride application with recall after three months Patient 2: indirect composite restoration with fluoride application Patient 3: composite restoration	Hypocalcified teeth should be treated with prosthetic rehabilitation, while hypoplastic teeth should be treated with direct composite restoration

**Table 3:** Case-control and cross-sectional studies on children with amelogenesis imperfecta.

Author(s)	Location	Study design	Cases	Main findings
Lafferty et al. (2022) [37]	United Kingdom	Cross-sectional	138	In the primary dentition, the use of composite to restore teeth was low. Preformed metal crowns (PMCs) and extractions were the preferred treatment options with wide standard deviations of 2.9 and 3.4, respectively. In the permanent dentition, anterior composite restorations were the most common treatment provided, with both PMCs and composites used to restore posterior teeth
Ohrvik et al. (2021) [38]	Norway	Retrospective study	154	All-ceramic enamel-dentin bonded restorations, prefabricated composite veneers, and direct composite resin restorations. Surface and color calibration showed a success of 95% for the ceramic enamel-dentin bonded restorations, 44% for the direct composite resin restorations, and 0% for the prefabricated composite veneers
Lyne et al. (2020) [39]	United Kingdom	Multicenter treatment evaluation study	60	Fissure sealants; stabilize teeth with direct restorations or preformed metal crowns; definitive restorations for anterior teeth. Children with full-mouth rehabilitation were more satisfied with the treatment
Gabardo et al. (202) [40]	Brazil	Case-control	27 AI patients; 27 controls	Radiographic evaluation of dental maturity of AI patients was delayed by around a year compared to healthy control
Quandalle et al. (2020) [41]	France	Case-control	42 AI patients; 42 controls	Participants with hypocalcified AI had more gingival inflammation, enamel defects, and tooth sensitivity than patients with the hypoplastic and hypomature subtypes
Yassin (2016) [42]	Saudi Arabia	Cross-sectional	Four patients with AI	AI is not common in children from the Aseer region of Saudi Arabia. Restorative treatment with indirect restoration was the treatment of choice
Sneller et al. (2014) [43]	United Kingdom	Exploratory study	Four patients with AI	Online support groups with pediatric dentists could help parents and children understand AI and discuss the proper management of AI according to its severity

**Table 4:** Review articles on amelogenesis imperfecta and restorative treatment.

Author(s)	Country	Study design	Main findings
Strauch and Hahnel (2018) [44]	Germany	Review	Dental treatment of amelogenesis imperfecta (AI) patients with direct composite restoration and periodontal scaling has provided outstanding results. Other treatments, including the adhesive bonding technique, have shown limited results with longevity of treatment outcomes. Hence, scientific evidence suggests indirect composite restoration over direct composite restoration
Shivhare et al. (2018) [45]	Nepal	Review	Minimal intervention glass ionomer cement restorations are advised in the primary dentition and direct and indirect composite resin veneers in the mixed dentition. However, no standard guidelines for treating AI patients have been developed
Smith et al. (2017) [46]	United Kingdom	Review	AI genetics can improve the clinical outcomes of various types of AI at the early stages, help clinicians plan treatment accordingly, and improve the quality of life of individuals suffering from AI
Sabandal and Schäfer (2016) [47]	Germany	Review	Comparison of restorative treatment longevity resulted in the best outcomes with hypoplastic AI compared to hypomature and hypocalcified AI. This result is due to less carious and fractured teeth associated with hypoplastic AI
Wallace and Deery (2015) [48]	United Kingdom	Review	Direct composite restoration is the treatment of choice in patients with hypoplastic AI. Porcelain veneers are indicated in patients with mixed dentition suffering from AI. However, it is contraindicated in deciduous dentition due to large pulp chambers
Alachioti et al. (2014) [49]	United Arab Emirates	Review	Direct composite restorations are strongly recommended for children and adolescents with AI, as they can be easily adjusted according to dentoalveolar development and are minimally invasive. Diagnosis and treatment of AI patients with anterior open bite require comprehensive treatment with multiple approaches. This will provide patients with a better quality of life and satisfaction
Dashash et al. (2013) [50]	Australia	Systematic review	Well-designed randomized controlled trials should be planned to determine which material is best for the clinical and esthetic outcomes of the patients suffering from AI

Collating and Summarizing the Findings

This scoping review condenses a significant amount of clinically relevant information on AI, encompassing various articles addressing its etiology, classification, and management. The main findings are outlined. AI should be clinically and radiographically differentiated from similar dental disorders such as dentogenesis imperfecta, dentin dysplasia, vitamin D rickets, tetracycline staining, fluorosis, and regional odontodysplasia. While it was initially postulated that AI is an isolated trait not associated with any syndrome, recent research has identified its association with nephrocalcinosis, also known as enamel-renal syndrome, resulting from mutations in the *FAM20A *gene. Genetics involved in the development of AI have not been identified; however, 10 genes have been associated with AI and are found in molecular diagnosis in most cases. In the past, children with AI were only given oral hygiene instructions and fluoride applications, with treatment initiated after the complete eruption of permanent teeth [19,20]. Contemporary approaches involve restorative and prosthetic treatments, along with strict dietary and oral hygiene instructions, serving as the primary line of treatment for AI in both children and adults. Additionally, all included studies suggested regular follow-up appointments to maintain good oral hygiene.

Reports From Case Reports and Series

Nineteen articles were included in this review reporting various AI cases and management in children aged between three and 14 years (Table 2). The management described in all cases reported is briefly described below.

The management of AI varies according to the age of dentition and is divided into three phases. Phase I is a temporary stage designed for primary and mixed dentition. Phase II, a transitional phase, occurs when all permanent teeth have erupted and continue into adulthood. Phase III, the permanent phase, involves treatment for fully developed permanent teeth. Studies suggest that dental treatment for children affected by AI should ensure favorable conditions for permanent teeth eruption and normal facial bone and temporomandibular growth [19,21,22,30]. The treatment is divided into two stages. In the first stage, anterior teeth are minimally invasively restored for discoloration using materials such as resin-modified glass ionomer cement, polycarbonate crowns, prefabricated crowns, and direct composite restorations [25,29,34]. In the second stage, fully erupted permanent molars are restored with metal-ceramic crowns to prevent caries development and the attrition of defective enamel. However, patients with severe forms of AI (hypomature and hypocalcified) face challenges as the adhesive strength of enamel and dentin is compromised. In such cases, overdentures are provided to maintain eruption and vertical dimensions of occlusions, improving the patient’s tolerance for future treatment. However, this approach requires diligent oral hygiene due to plaque accumulation in the gaps [32,33].

Hypomature AI enamel is prone to fracture due to thin enamel, cervical constriction, and thin roots. Similarly, dentine has a loose structure and lacks hardness, making intracoronal restorations contraindicated [25,26,32,34]. Pulpectomy has a poor prognosis due to root structure and calcified pulp stones in the roots. As a result, extraction is often necessary for hypomatured teeth with periapical infections. In less severe cases of AI, carbamide peroxide bleaching is also recommended [25,26,32,34].

In mixed dentition, the treatment goals primarily focus on preserving tooth structure, vitality, sensitivity, esthetics, and vertical dimensions. The first line of treatment, as indicated in various included studies, is orthodontic and prosthetic rehabilitation. Restorative rehabilitation in mixed dentition is a complex procedure because the definitive treatment cannot be provided until the eruption sequence of the teeth is completed. For permanent molars, stainless-steel crowns are commonly used to maintain the vertical dimension [19,21,33]. In addition, inlay and onlay restorations are preferred for preserving the occlusion of posterior teeth. Various restorative treatment modalities have been reported to enhance dental esthetics. The studies that were included suggest that indirect composite resin veneers are among the best restorative options for masking anterior teeth and improving crown morphology. Full-coverage adhesive composite resin or polycarbonate crowns are also advocated as treatment options for mixed dentition [19,21,33].

In permanent dentition, the treatment objectives are to reduce teeth sensitivity and restore vertical dimension, function, and esthetics. The final treatment commences as soon as the clinical height of the crown is established and the pulp tissue has receded. Full-mouth rehabilitation with a multidisciplinary approach is advisable at this stage, incorporating prosthodontic, endodontic, restorative, periodontic, and orthodontic management [19,20,21,26,28,33]. Some studies have suggested orthognathic surgery for abnormal skeletal growth. Crown lengthening and gingival recontouring are recommended in cases with short gingival crowns and hyperplasia [16,28,40]. Anterior open bite, often associated with AI, requires orthodontic treatment to correct. Root canal therapy is advised for patients with severe tooth attrition [27,33]. Consultation with specialists is essential for developing a comprehensive treatment plan for individuals suffering from different types of AI.

Reports From Observational Studies

Seven observational studies were included [37-43], encompassing various aspects of AI, ranging from prevalence to complex management (Table 3). Two studies from the United Kingdom examined the success of different treatment approaches for children with AI, emphasizing the necessity of a multidisciplinary approach. They concluded that preventive measures should constitute the primary line of treatment [37,40]. The retrospective study by Ohrvik et al. on restorative management in children with AI reported a 44% success rate with prefabricated direct composite resin restoration but noted a 0% success rate with prefabricated veneers [38]. The authors recommended further research in this area. Two case-control studies in the review indicated that dental maturity in children with AI is delayed compared to the control group.

Additionally, hypocalcified AI was seen to be associated with more dental anomalies than hypomature and hypoplastic AI [37,39]. An explanatory study proposed the establishment of online groups comprising parents, children, teenagers with AI, and pediatric dentists to better understand oral health issues related to AI and identify cost-effective treatment modalities [41]. A prevalence study by Yassin et al. reported that approximately 0.95% of children in the Aseer region of Saudi Arabia suffered from AI [42]. Overall, these findings underscore the importance of a comprehensive and collaborative approach to managing AI and highlight areas for further research.

Reports from Review Studies

This scoping review incorporates six reviews [44-49] and one systematic review [50] among the main findings of four reviews focused on the management of children with AI (Table 4). One review reported that indirect composite restorative management yields greater success in treating AI children than indirect restoration [44]. However, contrasting opinions emerged from two other reviews, suggesting that direct composite restoration is more successful than indirect methods [45,46]. Another review proposed that direct and indirect composite restoration demonstrate good long-term results for children with AI [47]. Furthermore, the reviews collectively emphasize the significance of primary prevention as the first line of treatment for AI children below the age of three years. The need for studies offering guidelines for managing AI in children was also highlighted. Dashash et al., in their systematic review, aimed to assess the best restorative material for AI treatment but noted the absence of a single randomized controlled trial evaluating the longevity of restorative materials [50]. The authors could not complete the review and recommended planning randomized controlled trials to assess the effectiveness of restorative materials for managing AI in children.

Discussion

This scoping review is the first to map and characterize the etiology, classification, and restorative treatment modalities of AI among children from a dental perspective. AI is a dental developmental disorder characterized by enamel defects and usually occurs without any underlying systemic disease [7]. AI comprises diverse phenotypes such as discoloration of teeth, taurodontism, and tooth malformation. The most common differential AI diagnoses include fluorosis, dentinogenesis imperfecta, enamel hyperplasia, and tetracycline stains [46]. AI can be diagnosed using clinical examination, radiological evaluation, and histopathological examination. Existing literature suggests that diagnosing rare genetic diseases should involve assessing genetic mutations and biochemical abnormalities and should consider family history [23,24,47].

In this scoping review, 75% of articles consisted of case reports and case series, indicating that AI is a rare disorder, and accumulating large samples for a clinical study is not feasible. Moreover, the prevalence of AI was recorded as 1:700 to 1:14,000, depending on the population of interest [7], with patients included in this review falling within the age group of three to 14 years. Treating patients within this age group is crucial, particularly for esthetic reconstruction and functional rehabilitation. This treatment is mandatory to prevent further damage to the teeth and disruptions in the vertical growth pattern. Most patients included in this review suffered from the hypoplastic type of AI (54%), followed by hypomatured (36%), and hypocalcified (10%). Similar findings were observed in other systematic reviews [48]. In this review, it was impossible to evaluate the prevalence of AI among the genders as, in most cases, gender was not mentioned. However, a study by Adorno-Farias et al. reported an equal prevalence of AI among the genders [49].

Pediatric dentists must comprehend the pathogenesis and clinical findings associated with various types of AI at an early stage. This will help them design proper treatment and protect teeth at an early age. The time required for managing AI in children varies based on the type and condition of the patient. Moreover, the patient’s cooperation with dental treatment is crucial, as it significantly influences the duration of the treatment. Timely prevention and corrective treatment can be achieved by following a few therapeutic measures, including (1) establishing rapport with family members and the patient; (2) re-establishing oral and masticatory functions while maintaining the vertical growth of facial muscles and alveolar bones; (3) preventing teeth from developing dental caries and periapical lesions leading to pain and sensitivity; (4) preserving the vitality, form, and size of the tooth; (5) maintaining proper eruption of permanent teeth; (6) decreasing the risk factors leading to tooth fractures; (7) maintaining the normal profile of an individual; and (8) preventing problems with temporomandibular disorders. Therefore, for AI patients’ early diagnosis, multidisciplinary approaches are required. The primary purpose of conducting this review is to obtain a prognosis and plan treatment because late intervention makes treatment complicated and time-consuming. Overall, the selection of restorative treatment and techniques for treating AI patients depends on the age and cooperation of the patient at the beginning of treatment, the parents’ consultation, and the severity of the disease. Due to long dental visits and the requirement of extensive dental procedures, pediatric dentists should consider using pharmacological sedation as a rationale for performing the treatment.

Literature suggests that the varying etiology of AI and a wide array of clinical problems make the restorative management of AI challenging for dentists [50,51]. As both the esthetic and function are compromised in AI patients, their management usually requires full mouth rehabilitation, depending on the condition and severity of the disease. AI patients often experience emotional trauma, leading to difficulties in eating, pain during brushing, social avoidance, and low self-esteem. The dental management of children with AI is challenging and requires a proper approach and comprehensive treatment strategy that outlines future treatment needs [31,37,42]. Pediatric dentists assisting children with AI should understand the clinical and psychological demands of the condition. This understanding is crucial to ensure that the extended procedures associated with AI treatment are manageable for children and that the disease does not burden them [31].

This scoping review has several limitations. First, the age limit considered by the author is from three to 14 years, a period during which the maturation of teeth does not occur. Hence, a proper management approach cannot be decided. This decision can be justified because the early treatment approach in patients with AI helps plan future treatment. Second, the articles were presented only in the English language. This can be justified because studies demonstrating the treatment of diseases are mostly published in English and cited by studies in other languages or converted into various languages [52,53]. Therefore, studies published in English are mostly cited and included in narrative, systematic, and scoping reviews; this is likely to introduce a bias (reductionism) [52,53]. While there is a chance that some articles and case reports in other languages may have been missed, the author is confident that the inclusion of substantial articles published in high-quality journals makes this scoping review comprehensive.

In conclusion, pediatric dentists should prioritize early diagnosis and treatment and long-term follow-up for AI in children to effectively enhance the patient’s psychological well-being and overall quality of life. The selection of a treatment approach should be based on an individual’s specific functional, cosmetic, restorative, and occlusal needs. However, the severity of tooth structure damage remains the primary factor in determining the treatment choice, and successful management results in improved oral hygiene and patient nutritional needs. High motivation from children and their parents is required to maintain a preventive approach, as they need to adhere to and follow all instructions.

## Conclusions

AI is a group of inherited genetic disorders that pose significant challenges in diagnostic and restorative dental treatment. Currently, no gold standard or guidelines are available for managing patients with AI. However, multidisciplinary approaches have shown favorable outcomes, particularly in children. There is a critical need for cumulated evidence on outcomes supporting restorative and conservative treatment across different types of AI. With proper guidelines, clinicians can plan comprehensive treatment for children with AI, optimizing their oral health and long-term prognosis. Therefore, it is highly recommended to plan and develop research aiming to compare the treatment planning, restorative materials utilized, and techniques for restoring the teeth of AI patients, which will eventually help develop a guideline for treatment planning.
